# The effect of aging on semen parameters in normozoospermic men: A cross-sectional study

**DOI:** 10.18502/ijrm.v20i11.12363

**Published:** 2022-12-10

**Authors:** Mahmut Ulubay, Muhammet Bahaettin Ulu, Ekrem Akdeniz

**Affiliations:** ^1^Department of Urology, School of Medicine, Samsun University, Samsun, Turkey.; ^2^Department of Urology, Gazi Hospital, Samsun, Turkey.; ^3^Department of Urology, Samsun Training and Research Hospital, Samsun, Turkey.

**Keywords:** Age, Fertility, Semen, Semen quality.

## Abstract

**Background:**

Semen parameters change with age and are reported differently worldwide.

**Objective:**

This retrospective cross-sectional study aimed to investigate the semen quality pattern among aging men and the age thresholds for semen parameters.

**Materials and Methods:**

The records of men who had normal semen parameters from January 2015-June 2020 were retrospectively evaluated for andrological outpatient at Samsun Training and Research hospital and Gazi hospital in Samsun, Turkey. Adult men meeting the inclusion criteria were divided into 3 groups of I) 18-29 yr (n = 629), II) 30-39 yr (n = 775), and III) 40-49 yr (n = 190). Correlations between age and sperm parameters were then analyzed.

**Results:**

A total of 1594 men were enrolled in the study. Significant differences were observed in total sperm numbers, total motility rates, progressive motility rates, nonprogressive motility rates, normal morphology rates, mean semen volume, and sperm concentrations. The parameters of total sperm number, progressive motility rate, and normal morphology rate were significantly higher in group I than in the other 2 groups (p < 0.001, p < 0.001, and p < 0.001, respectively) and in group II compared to group III (p = 0.001, p = 0.003, and p < 0.001), respectively. Mean semen volume and total motility rate were significantly higher in group I than in the other groups (p = 0.001 and p < 0.001, respectively). However, no difference was observed between group II and group III (p = 0.61 and p = 0.04, respectively).

**Conclusion:**

Age has a significant impact on semen parameters capable of affecting male fertility, particularly total sperm numbers, the progressive motility rate, and the normal morphology rate.

## 1. Introduction

Aging is an inescapable process resulting in various physiological changes. Individuals get married and plan to have children later in life, including their career, education, and economic and social factors (1). Various changes can occur in semen parameters with aging, for reasons such as stress, an unhealthy diet, decreased physical activity, chemical additives, psychological factors, and exposure of the scrotum to radiation in association with frequent use of technological devices, including laptop computers and cell phones (2-4).

Semen analysis is the first laboratory test performed for evaluating male fertility capacity. The world health organization manual (6 ${}^{\text{th}}$ Ed, 2021) cites the following semen parameter cut-off values: semen volume ( > 1.4 mL), total sperm count ( > 39 million), sperm concentration ( > 16 million/mL), motility ( > 30% progressive motile), and morphology ( > 4% normal forms) (5).

Research into the impact of paternal age on semen parameters is still inconclusive. Some studies have suggested that advanced paternal age can adversely impact sperm motility, vitality, and normal morphology (6-12). While, other studies indicated no significant age-dependent change in sperm parameters (13-15). However, most of the studies have not discriminated results on pathological and non-pathological semen analysis, and have not excluded potential diseases capable of affecting semen parameters, such as varicocele, undescended testis, or history of surgery. Only normozoospermic men were included in the present study, and pathologies capable of impacting semen parameters, such as varicocele, undescended testis, orchiopexy, and torsion, were excluded.

To the best of our knowledge, this study is unique from that perspective. In addition, there has been no research on this subject in Turkey. The study aimed to investigate the relationship between age and semen parameters (semen volume, total number, concentration, motility, progressive motility, and morphology) in normozoospermic men presenting due to infertility and undergoing computer-assisted semen analysis (CASA).

## 2. Materials and Methods

Semen analysis results for men presenting to departments for andrological outpatients at the Samsun Training and Research hospital and Gazi hospital in Samsun, Turkey due to infertility between January 2015 and June 2020 were evaluated in this retrospective cross-sectional study.

### Participants and evaluation

The study included 1594 men with normal semen analysis results presenting due to infertility. These were divided into 3 groups based on age: group I) 18-29 yr, group II) 30-39, and group III) 40-49 yr. Men with pathological semen analysis results, varicocele, undescended testes, or a history of orchiopexy, testis surgery, or solitary testis (since this would affect the semen analysis findings) were excluded from the study.

### CASA

Semen samples obtained by masturbation following abstinence of 3-7 days were directly placed into a sterile plastic container and analyzed in line with 1 hr. The world health organization 2021 guideline was used as a reference when evaluating semen parameters (5). The specimens were confirmed as semen samples at the macroscopic examination. Following liquefaction, samples were placed in an incubator for approximately 30 min at 37 C. Semen analysis was conducted on an SQA-V Gold sperm analyzer (Medical Electronic Systems Ltd. Caesarea Industrial Park, IL 3088900, UK) based on the laboratory-based CASA system in line with the manufacturer's instructions. The samples were first mixed thoroughly and placed into the electro-optic chamber of the apparatus with a capillary for CASA counting. The computer system contains special algorithms involving the translation of light beams into electrical signals to automatically report sperm counts and movements. Age, pH, ejaculate volume, total sperm number, sperm concentration, sperm morphology, total motility, progressive motility, and nonprogressive motility were analyzed in all 3 groups.

### Ethical considerations

All procedures involving human participants were conducted in strict compliance with the ethical principles of the Institutional Research Committee and the 1964 Declaration of Helsinki and subsequent modifications or equivalent ethical standards. This study was approved by the Samsun Training and Research hospital Medical Ethical Committee in Samsun, Turkey (Code: 33646832-799).

### Statistical analysis

Data analysis was performed using SPSS version 25 (Statistical Package for Social Sciences- IBM Corp., Armonk, NY, USA) software. The Kolmogorov-Smirnov test was applied to determine the normality of measurable data. Numerical variables were expressed as median values (interquartile range: 25 ${}^{\text{th}}$ -75 ${}^{\text{th}}$ percentile). Statistically significant differences among the different study groups were evaluated with the assistance of the Kruskal-Wallis test. A p-values < 0.05 were regarded as statistically significant. The Bonferroni-corrected Mann-Whitney U test was employed to determine the source of significance in variables identified as significant.

## 3. Results

**Figure 1:**
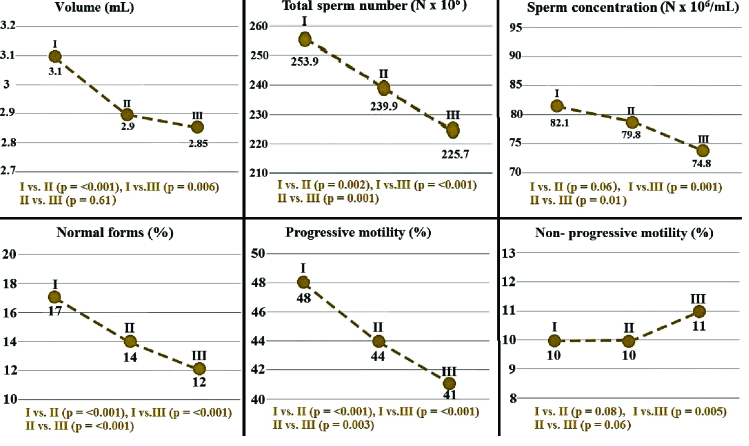
Semen parameter values among groups (I: Group I, age range: 18-29, n = 629, II: Group II, age range 30-39, n = 775, III: Group III, age range 40-49, n = 190).

The study was conducted with 1594 participants with a mean age of 32 yr (IQR: 27-36). The mean ages in groups I, II, and III were 26 (IQR: 24-28), 34 (IQR: 32-37), and 42 (IQR: 41-46) yr, respectively. No difference was observed among the groups regarding semen pH values (p = 0.37). As shown in table I, mean semen volume and total motility rate values differed significantly between the groups. The groups' age and semen characteristics are listed in table I. Semen parameters were significantly higher in group I than in the other 2 groups. However, no difference was determined between groups II and III. Intergroup analysis for semen parameters in different groups is shown in figure 1.

As shown in table I, total sperm numbers, progressive motility rates, and normal morphology rates also differed significantly between the groups (p < 0.001). All 3 parameters were significantly higher in group I than in groups II and III (Figure 1). The highest sperm concentration value was observed in group I, and the highest nonprogressive motility rate in group III, being both elevations statistically significant (Table I). Intergroup analysis revealed a significant difference only between groups I and III, other differences being insignificant.

**Table 1:** Ages and semen parameters in the study groups


**Variables**	**Group I (18-29 yr)**	**Group II (30-39 yr)**	**Group III (40-49 yr)**	**P-value**
**Number**	629	775	190	
**Age (yr) **	26 (24-28)	34 (32-37)	42 (41-46)	< 0.001
**pH**	8.5 (8-9)	8.5 (8.5-8.5)	8.5 (8-8.5)	0.37
**Volume (mL)**	3.1 (2.2-4.1)	2.9 (2.2-3.7)	2.85 (2.2-3.4)	0.001
**Total sperm number (x 10^6^)**	253.9 (187.7-334.4)	239.9 (151-323.6)	225.7 (178.62-260.4)	< 0.001
**Sperm concentration (x 10^6^/mL) **	82.13 (53.85-127.05)	79.83 (54.51-117.68)	74.8 (47.44-100.8)	0.003
**Normal morphology (%)**	17 (11.5-20.5)	14 (9-19)	12 (9-16)	< 0.001
**Total motility (%)**	58 (52-66)	55 (48-61)	53 (47-60)	< 0.001
**Progressive motility (%)**	48 (41-56)	44 (38-51)	41 (37-49)	< 0.001
**Nonprogressive motility (%)**	10 (8-13)	10 (8-13)	11 (8-14.25)	0.014
All data expressed as median and interquartile range. Kruskal-Wallis				

## 4. Discussion

The present study involved the analysis of an extensive set of semen parameters to investigate the effect of aging. Total sperm counts, normal morphology rates, and progressive motility rates decreased with age, with a concomitant increase in nonprogressive motility. The feature distinguishing the present study from previous research is that we enrolled only normozoospermic men. Men with abnormal sperm parameters or diseases capable of affecting semen analysis results were excluded even if they were normozoospermic (varicocele, undescended testis, trauma, solitary testis, or orchiopexy). These features distinguish the present study from the previous research.

One feature of the modern age is that men are increasingly becoming fathers later in life. The number of men becoming fathers between the ages of 35 and 54 in the UK has increased by 15% compared to 10 yr ago (16). The average age at fatherhood in Denmark in 1986, 30.9 yr, had risen to 33.4 by 2016 (12). This increases the importance of sperm quality in the aging male, and the number of studies investigating the relationship between age and sperm quality has grown in recent years (6-21).

Figure 1 shows the different semen parameters analyzed in this study. The findings indicate that total sperm count, sperm morphology, and progressive motility decreased significantly from the 20s to 40s. Other studies have also reported that total sperm count and progressive motility decreased with age. However, in contrast to the present study, those authors observed no statistically significant correlation between age and sperm morphology (8, 12). In one such study, the probability of a decreased sperm count was 2.92-fold greater among men aged between 41-50 compared to those aged between 21-30 yr, while men aged > 50 were 11.91 times more likely to exhibit impaired sperm progressive motility than men aged between 21-30 (8).

Similar to the present research, another study divided the participants into 3 age groups and also reported significant decreases in progressive motility and sperm morphology (17). In contrast to the present study; however, those authors reported similar total sperm numbers between the age groups 29-39 and 40 or higher, while numbers in the 18-28 age groups were significantly higher. Interestingly, the total sperm count in the age group 29-39 was lower than those in their 40 and over, although the difference was not statistically significant (17). A separate piece of research noted that progressive motility began decreasing significantly from the age of 28, and total sperm numbers from the age of 42 (7). However, in contrast to these studies and the present research, other authors have observed no difference in total sperm count, sperm morphology, or total motile count between men under and over 40 (18).

Although most studies have reported an inverse correlation between age and semen volume, there is still no consensus on this subject. In one extensive study, the researchers observed that semen volume diminished with age, and that the risk of decreased semen volumes increased by 1.06-fold compared to the previous year (8). 2 other studies reported significant age-dependent decreases in semen volume (12, 18). In contrast, other authors have reported no correlation between age and semen volume (7, 17, 19). However, semen volume was significantly higher in the age group 18-29 than in the other 2 groups, while no significant difference was observed between the age groups 30-39 and 40-49. No significant relationship was observed between aging and semen volume. There appears to be a definite relationship between age and semen volume in the literature. Therefore, we conclude that factors capable of affecting semen volumes, such as diet, heredity, overall health, and smoking status, should be excluded when investigating the relationship between semen volume and age.

Inconsistent findings have also been reported regarding the relationship between aging and sperm concentration. According to 2 previous studies, sperm concentration increases significantly with age (18, 20). However, other studies have reported the opposite (7, 8). No association between sperm concentration and age was determined in a systematic review of 12 studies or other extensive studies (12, 21). Only normozoospermic men were included in the present study, and individuals with testis pathologies were excluded even if their semen parameters were normal. However, these 2 studies did not adopt the broad exclusion criteria employed in the present study, with all men being included, even those with semen parameter impairment. We think that this accounts for the differences between our studies. A significant decrease in sperm concentration was observed only between the age groups 18-29 and 40-49 in the present study. Although the findings in the literature are inconsistent, the results concerning the relationship between aging and sperm concentration are compatible with one previous study (17).

The authors of a blind cross-sectional study involving 11,706 participants, described age as an adverse factor in terms of sperm volume, sperm motility and vitality, and sperm kinematic variables, and that, together with age, clinical conditions such as cancer and cardiovascular and respiratory diseases, significantly exacerbated this worsening. The authors also reported that obesity resulted in less pronounced damage to sperm quality, while cigarette smoking and alcohol consumption emerged as more harmful than obesity (12).

One study team evaluated 12,538 cases of oocyte donation and reported that semen volume decreased with age. These authors attributed this to decreased androgen-stimulated fluid production in the prostate and seminal vesicles due to an age-related decrease in androgen levels. They also reported that sperm motility decreased with age, indicating an age-related diminution in epididymal and accessory sex gland functions. No age-related change in sperm concentration or morphology was observed. The basic aim of these studies was to determine the effect of paternal age on oocyte donation outcomes. Research has concluded that there is no association between advancing paternal age and adverse oocyte donation outcomes, including pregnancy and live-birth rates (21).

There are several limitations to this study, including its retrospective nature. However, the most important limitation is that individual factors capable of damaging sperm, such as smoking, infections, hormone levels, diet, or chronic alcoholism, were not included due to the unavailability of data. These might have impacted our results. Further prospective, multicenter studies including parameters such as smoking, infections, hormone levels, diet, and chronic alcoholism are now required to confirm these findings.

## 5. Conclusion

Age is negatively correlated with semen parameters, especially total sperm number, progressive motility rate, and normal morphology rate, in normozoospermic men with no testis alterations capable of impairing semen parameters. It should not be forgotten that while semen parameters are not absolute evidence of male fertility potential, they nevertheless decrease with age, and the age factor should be considered in the treatment of infertile couples.

##  Conflicts of Interest

The authors declare that there is no conflict of interest.
